# Mechanically Enhanced Ultrashort Peptide Hydrogels for pH-Triggered Release

**DOI:** 10.1021/acspolymersau.6c00020

**Published:** 2026-04-16

**Authors:** Pasqualina Liana Scognamiglio, Carlo Diaferia, Mariantonietta Pizzella, Antonella Accardo, Giancarlo Morelli, Diego Tesauro

**Affiliations:** † Department of Basic and Applied Sciences, University of Basilicata, 85100 Potenza, Italy; ‡ Department of Pharmacy and Interuniversity Research Centre on Bioactive Peptides (CIRPeB), University of Naples “Federico II”, 80131 Napoli, Italy; § 591458 IRCCS, SYNLAB SDN, 80146 Napoli, Italy

**Keywords:** supramolecular self-assembly, peptide-based hydrogels, stimuli-responsive materials, pH-Responsive hydrogels, drug delivery

## Abstract

Ultrashort peptide-based hydrogels represent an attractive class of supramolecular soft materials due to their minimalistic design, chemical versatility, and potential for translational applications. Classical Fmoc-dipeptides, particularly Fmoc-FF, are well established as efficient low-molecular-weight hydrogelators; however, controlling their aqueous solubility and gelation behavior across physiologically relevant conditions remains a key design challenge for injectable and in situ forming materials. Here, we report a pH-responsive injectable hydrogel based on a dipeptide incorporating the unnatural amino acid Fmoc-β-(3-pyridyl)-l-alanine (Fmoc-3-Pal-OH). Introduction of the pyridyl moiety provides a well-defined protonation–deprotonation equilibrium that acts as a molecular switch to regulate the supramolecular self-assembly. Deprotonation in the pH range 6.0–8.0 promotes spontaneous hydrogel formation under mild conditions, while protonation at acidic pH induces a controlled network disassembly. The hydrogel system was comprehensively characterized as a function of the concentration and buffer conditions using fluorescence spectroscopy, circular dichroism, Fourier-transform infrared spectroscopy, scanning electron microscopy, and rheology. pH modulation enables fine control over nanofibrillar organization and viscoelastic properties, yielding mechanically stable hydrogels with tunable stiffness values comparable to those of soft biological tissues. The pH-dependent assembly behavior was further exploited to regulate drug release. Encapsulation of curcumin as a hydrophobic model compound demonstrated high loading capacity and sustained release under neutral conditions, while acidic pH triggered an accelerated release through protonation-induced network collapse. Overall, the results achieved by this research open the way to a simple and robust molecular design strategy to overcome key limitations of conventional Fmoc-based hydrogelators and highlight the potential of protonation-controlled ultrashort peptide assemblies as adaptable polymer-like networks for stimuli-responsive soft materials and drug delivery applications.

## Graphical abstract

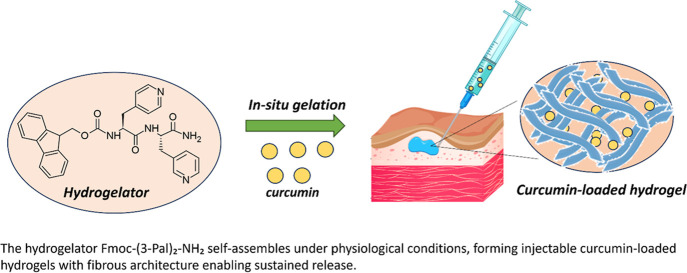

## 1 Introduction

Hydrogels are a versatile class of soft materials, widely valued for their broad applicability, thanks to their high water content, tunable mechanical properties, and excellent biocompatibility.^1–3^ Among them, supramolecular or self-assembling hydrogels have attracted increasing attention for applications in wound healing, tissue engineering, drug delivery, diagnostics, and biosensing, owing to their dynamic and reversible nature, biocompatibility, and ability to mimic the extracellular matrix.^4–6^ Within this context, peptide-based hydrogels have emerged as a particularly promising subclass, due to their efficient self-assembly, structural versatility, and molecular tunability.^7,8^ These materials are known to self-assemble under physiological conditions through noncovalent interactions such as hydrogen bonding, π–π stacking, hydrophobic interactions, and electrostatic forces. Easily tunable through external stimuli, such as pH changes, temperature shifts, or sonication, peptide hydrogels can respond dynamically to their environment and encapsulate a variety of drug molecules.^9^ Their properties are strongly influenced by the peptide sequence and resulting secondary structures, allowing precise control over gelation behavior and expanding their potential applications.^10,11^ Hydrogels based on di- or tripeptides represent a particularly interesting subset of these systems.^12,13^ Among them, diphenylalanine (-FF) is one of the most extensively studied dipeptides, with self-assembly largely driven by the aromatic interactions between the two phenyl rings. These short peptides have garnered significant interest due to their inherent ability to organize into diverse nanostructures, such as fibers, rods, ribbons, and nanotubes, which can be further tailored by sequence modification or functionalization of side chains.^14–17^ The incorporation of synthetic end-capping groups, like naphthalene, fluorenylmethyloxycarbonyl (Fmoc), or anthracene, into dipeptides has been shown to enhance their gelation ability and mechanical strength.^10,18–20^ Among these, Fmoc-Phe-Phe stands out as a particularly efficient hydrogelator, and it has been extensively studied since 2006 for its broad applicability. Jayawarna et al. demonstrated that the gelation behavior of Fmoc-dipeptides (composed of glycine, alanine, leucine, and phenylalanine) varies significantly with sequence and pH. Notably, Fmoc-Phe-Phe forms hydrogels at pH < 8, while most others gel only at pH < 4, and some, like Fmoc–Gly–Phe, fail to gel entirely.^21^ These findings highlight the critical role of amino acid composition and sequence in determining the hydrogel properties. Thus, most of these dipeptides typically form hydrogels at low pH, which can significantly limit their biomedical applications, as such conditions are often incompatible with physiological environments and may affect cell viability and biomolecule stability.^22,23^ This restricts their use in vivo, particularly for injectable or implantable systems where mild, body-like conditions are essential. In contrast, peptide-based gelators capable of forming in situ hydrogels under physiological conditions provide a valuable alternative. Moreover, their ability to gel under biocompatible conditions enables controlled drug release, minimally invasive parenteral delivery, and broadens their applicability to advanced biomedical technologies such as 3D printing and tissue engineering.^24^ These hydrogels generally exhibit low mechanical strength because of their supramolecular nature, with storage moduli typically not exceeding 1000 Pa. Consequently, their limited mechanical stability may compromise the integrity of the hydrogel network and affect the retention and controlled release of the loaded drug. To date, only a few mechanically strong dipeptide hydrogels have been reported.^25,26^ In most cases, enhanced mechanical performance is achieved through hybrid strategies, often involving reinforcement with carbon nanotubes or long alkyl chains to reinforce van der Waals interactions.^25,27^ Despite their potential, ultrashort peptide hydrogels still face key limitations, such as slow gelation, low drug loading, weak self-healing, and safety concerns that restrict their biomedical use. Hence, the development of novel hydrogels with stronger noncovalent interactions could enhance the retention and controlled release of therapeutic agents, while the use of biocompatible, biodegradable, and stimuli-responsive systems may further improve release kinetics, enable in situ gelation, increase therapeutic efficacy, and reduce side effects. Among the various external stimuli employed to trigger hydrogel formation, pH represents one of the most widely used and effective approaches. Variations in pH can modulate the protonation state of ionizable functional groups, thereby altering the balance between hydrophilic and hydrophobic interactions that govern the self-assembly processes. As a result, pH changes can induce sol–gel transitions and enable fine-tuning of both the structural organization and mechanical properties of peptide-based hydrogels. This responsiveness makes pH-sensitive systems particularly attractive for biomedical applications, including controlled drug delivery and tissue engineering.^28–30^ Herein, we designed a novel acid-sensitive hydrogel based on an analogue of Fmoc-FF, namely, Fmoc-(3-Pal)_2_-NH_2_, obtained by incorporating the unnatural amino acid Fmoc-β-(3-pyridyl)-l-alanine (Fmoc-3-Pal-OH). Recent studies have highlighted the versatility of this building block in tuning supramolecular architectures and functional properties in peptide-based materials. Notably, Gazit et al. demonstrated that Fmoc-(3-Pal)-OH exhibits supramolecular helicity with defined handedness, as well as well-organized self-assembled structures with notable mechanical and piezoelectric properties.^31^ The incorporation of the pyridine moiety provides additional functionality, as its protonation–deprotonation behavior enables pH-responsive properties and enhances aqueous solubility under acidic conditions. This feature has also been exploited in biologically active peptides, where substitution with pyridine-containing residues preserved the biological activity of glucagon analogues.^32^ Dipeptide-based systems were selected over single amino acid derivatives to ensure the enhanced structural stability and mechanical robustness of the resulting hydrogels. In this context, Fmoc-(3-Pal)_2_-NH_2_ combines the strong self-assembling propensity of dipeptides with the pH-responsiveness of the pyridine ring, enabling hydrogel formation under physiological conditions. The self-assembly and gelation processes of Fmoc-(3-Pal)_2_-NH_2_ were optimized by characterizing the structure and morphology of the system through a combination of techniques, including circular dichroism (CD), Fourier-transform infrared (FT-IR) spectroscopy, fluorescence and UV–Vis spectroscopy, rheology, scanning electron microscopy (SEM), and transmission electron microscopy (TEM). In addition, preliminary cellular biocompatibility studies confirmed that the material is well tolerated under the tested conditions, further supporting its potential for biomedical applications. As a proof of concept, we investigated the in vitro release of curcumin, demonstrating the ability of these nanostructured peptide hydrogels to act as versatile and stimuli-responsive platforms for controlled drug delivery.

## 2 Experimental Section

### 2.1 Materials

All chemicals were purchased from Merck (Milan, Italy), Fluka (Bucks, Switzerland), or LabScan (Stillorgan, Dublin, Ireland) and were used as received unless otherwise stated. The ^1^H NMR spectrum was acquired with Bruker 400 MHz and ^13^C NMR was acquired with Bruker 100 MHz. The identity of the peptide was assessed by mass spectrometry using a LTQ XL Linear, ion trap mass spectrometer, electrospray ionization (ESI) source (Finnigan/Thermo Electron Corporation).

### 2.2 Synthesis and Characterization of the Dipeptide Fmoc-(3-Pal)_2_- NH_2_

The dipeptide Fmoc-3 Pal-3 Pal-NH_2_ (Fmoc-(3-Pal)_2_-NH_2_) was synthesized in the solid phase using the well-assessed Fmoc strategy. Briefly, a rink amide MBHA resin (0.65 mmol/g, 77 mg, 100 μmol) previously treated with 30% piperidine in DMF to remove the Fmoc protection was used. Subsequently, Fmoc-3 Pal-OH (200 μmol, 2 equiv), DIEA (500 μmol, 5 equiv), HBTU (500 μmol, 2 equiv), HOBt (500 μmol, 2 equiv) were dissolved in DMF, added to the resin, and left under stirring for 30 min. The resin was then washed with DMF and treated with piperidine in DMF for the Fmoc deprotection. We repeated the coupling step with Fmoc-3 Pal-OH in the same condition to obtain the final product. Finally, the dipeptide Fmoc-(3-Pal)_2_-NH_2_ was obtained pure as a white solid (91% overall yield) after treatment with TFA/TIS/H_2_O (95/2.5/2.5, v/v/v) over 2 h, followed by precipitation from cold diethyl ether, centrifugation, and final lyophilization. The dipeptide was purified by reversed-phase HPLC. The method used consisted of a mixture of acetonitrile/water with 0.05% trifluoroacetic acid, with a flow of 10 mL/min, and solvent gradient: 0–3 min 1% acetonitrile, 16 min 60% acetonitrile using a C-18 column (25 × 21.2 mm, Agilent).

^1^H NMR (400 MHz, DMSO-*d*_6_) δ 8.70 (d, 2H), 8.68 (d, 2H), 8.28 (d, 2H), 7.88 (d, 2H), 7.78 (m, 1H), 7.70 (m,1H), 7.58 (m, 2H), 7.42 (dd, 2H), 7.3 (m, 2H), 4.6 (m, 1H), 4.3 (m, 1H), 4.20–4.07 (m, 3H), 3.22 (dd, 1H), 3.12 (dd, 1H), 2.98 (dd, 1H), 2.90 (dd, 1H),

^13^C NMR (100 MHz DMSO-*d*_6_) 172.3, 171.2, 156.2. 144.3, 141.0, 128.1, 127.5, 125.8, 120.6, 66.3, 55.8, 53.2, 47.1, 35.2, 34.4.

ESI-MS: C_31_H_29_N_5_O_4_, 535.22 Da (*m*/*z* + 1) with a retention time of 8.2 min.

### 2.3 Spectroscopic Characterization: Fluorescence and Circular Dichroism

Fluorescence experiments were performed on a Jasco spectrofluorophotometer (Model FP-750) at 25 °C. For these measurements, the dipeptide samples were prepared in 0.1 mol L^–1^ sodium phosphate (pH 8.0) at concentrations ranging from 5.0 × 10^–3^ to 1.6 × 10^–1^ mg mL^–1^. All of the fluorescence spectra were recorded in the 275–400 nm (λ_em_) range upon excitation at 260 nm (λ_ex_), using 5 nm slit bandwidths for both excitation and emission and finally corrected for background fluorescence by subtracting the proper buffer blank. All experiments were performed in duplicate. Fluorescence was also monitored over time for up to 30–60 min, keeping the peptide concentration constant at 0.16 and 0.32 mg mL^–1^. All fluorescence spectra were recorded in the 280–400 nm emission range upon excitation at 260 nm, using 5 nm slit bandwidths for both excitation and emission. Spectra were corrected for background fluorescence by subtracting the appropriate buffer blank. All experiments were performed in duplicate. The determination of CAC (Critical Aggregation Concentration) values was determined as previously described^33^ by the titration of the fluorescent probe ANS (8-anilino-1-naphthalene sulfonic acid ammonium salt) with the dipeptide. In detail, a solution of ANS in the cuvette (2.0 × 10^–5^ M) was titrated with several concentrations of Fmoc-(3-Pal)_2_-NH_2_ and spectra recorded between 290 and 500 nm, after excitation of samples at 280 nm. The fluorescence intensities maximum measured at 470 nm have been plotted as a function of peptide concentration. Far-UV CD spectra were recorded on a Jasco J-810 spectropolarimeter equipped with a Neslab RTE111 thermal controller unit at 25 °C. Fmoc-(3-Pal)_2_-NH_2_ solutions were prepared in 0.1 mol L^–1^ sodium phosphate (pH 8.0) in the 0.5 ÷ 5.0 mg mL^–1^ concentration range. Spectra were acquired by averaging three scans in the 195–260 nm range with a 2 nm bandwidth and using a 100 nm min^–1^ scan rate. All of the spectra were corrected for the background by subtracting the proper blank.

### 2.4 Hydrogel Formulation

Hydrogels were prepared by using a standard dilution approach. Briefly, stock solutions of the lyophilized peptide were prepared by dissolving the compound in Milli-Q water at a concentration of 30 mg mL^–1^. These stock solutions were then diluted with phosphate buffer (pH 8.0 and 6.0) to obtain final peptide concentrations of 0.1, 0.5, 1, 5, and 10 mg mL^–1^. The resulting mixtures were sonicated for 30 s and incubated at room temperature until the formation of a clear, self-supporting hydrogel. Hydrogel formation was confirmed by the vial inversion (tilt) method. The degree of protonation of the pyridyl nitrogen at each pH was calculated using the Henderson–Hasselbalch equation (p*K*_a_ ≈ 5.3) as follows

$$fraction\;protonated=\frac{1}{1+10^{\left(pH-pKa\right)}}$$

The corresponding percentages of protonated and deprotonated species are reported in the main text.

### 2.5 Fourier Transform Infrared Spectroscopy

Fourier Transform Infrared spectra of Fmoc-(3-Pal)_2_-NH_2_ at a concentration of 10 mg mL^–1^ was collected at the solid state on the spectrometer Jasco Fourier-transform /IR 4100 (Easton, MD) in the ATR mode, using a Ge single-crystal at a resolution of 4 cm^–1^. All of the spectra (100 scans with a rate of 2 mm·s^–1^ against a KBr background) were collected in the transmission mode and then converted in emission.

### 2.6 Scanning Electron Microscopy and Transmission Electron Microscopy

The morphological characterization of our hydrogels was carried out by SEM analysis through the collection of images using a Phenom Pro X G6, Netherlands, 450-FEI microscope at 5 kV. For sample preparation, 20 μL (diluted in phosphate buffer pH 8.0, 0.1 mol L^–1^) of the preformed hydrogel were deposited on a thin glass slide and air-dried for 16 h at room temperature. A 7 nm gold coating was applied via sputtering using a LUXORau SEM coater (Aptco Technologies, Nazareth, Belgium) at a current of 25 mA for 75 s. The sputter-coated samples were then transferred to the specimen chamber, and images were acquired at an accelerating voltage of 15 kV by using the Secondary Electron Detector (SED).

TEM analyses were performed on 1.0 mg mL^–1^ peptide solutions prepared at pH 6.0 and pH 8.0. A small aliquot of each sample was deposited onto carbon-coated copper grids and allowed to adsorb for 1 min. Excess liquid was removed by gentle blotting, and the grids were negatively stained with a freshly prepared aqueous solution of phosphotungstic acid (2% w/v, pH adjusted to ∼7). Micrographs were acquired using a FEI TecnaiG2 200 kV transmission electron microscope with an accelerating voltage of 120 kV. Fibril diameters were measured using ImageJ, yielding average values of 12–14 nm under both pH conditions.

### 2.7 Rheological Characterization

Rheological measurements were performed on a Malvern Kinexus instrument equipped with a 15.0 mm flat-plate geometry PU20/PL61 at 25 °C. A parallel plate geometry was used (final gap size of 0.60 mm). Strain sweeps (0.01–100%) were conducted to determine the linear viscoelastic region. The analyses were also conducted at different temperatures increasing from 25 to 80 °C. Amplitude (γ) was set at 0.1% and frequency = 1 Hz. *G*′ (storage modulus) and *G*″ (loss modulus) are reported as functions of the temperature. The self-healing property was assessed via step-strain measurements at constant frequency (ν = 1.0 Hz) starting from small-amplitude strain (γ = 0.1%, 60 s) followed by a 30 s large amplitude (γ = 80%) in a two-cycle experiment.

### 2.8 Stability Studies

Preformed hydrogels (100 μL) were incubated with 500 μL of two different 0.1 mol L^–1^ buffer solutions: a phosphate buffer at pH 7.4 and an acetate buffer at pH 4.5. At each time point, from day 0 to day 10, the residual mass of the hydrogel was carefully measured after removing the supernatant. The residual mass was then normalized to the initial mass to calculate the percentage of mass retention, providing a quantitative assessment of hydrogel stability and disassembly kinetics as a function of pH.

### 2.9 Cell Cultures and In Vitro Toxicity Assay

Human Embryonic HEK293 Kidney (HEK-293) cells were cultured in Dulbecco’s modified Eagle Medium (DMEM) (Gibco, Thermo Fisher Scientific Waltham, Massachusetts, US), supplemented with 10% fetal bovine serum (FBS) (Gibco), 2 mM glutamine, 100 U mL^–1^ penicillin, and 100 μg mL^–1^ streptomycin (Gibco) at 37 °C in 5% CO_2_. Cell confluence was maintained at 60–80%, and subculturing was performed at a 1:3 ratio twice a week using 25 cm^2^ flasks (Nunc, Thermo Fisher Scientific, US). For the toxicity assay, cells were seeded in 96-well plates at a density of 5.0 × 10^5^ cells per well 24 h before the treatment. Cells were then incubated for 48 h at 37 °C in the presence of conditioned media, according to the protocol previously described.^34^ At the end of the treatment, cell viability was assessed by the MTT (3-(4,5-dimethylthiazol-2-yl)-2,5-diphenyltetrazolium bromide) assay, according to the manufacturer’s instructions. Briefly, 20 μL of 5 mg/mL MTT solution in PBS were added to each well and then incubated for 4 h at 37 °C, 5% CO_2_. Absorbance of the dark blue formazan crystals, depending on the cell viability, was measured with a spectrophotometer (λ_max_ = 570 nm). All the analyses were performed in triplicate. Cell survival was expressed as a percentage of viable cells in the presence of hydrogels compared to control cells grown in their absence.

### 2.10 Formulation of Curcumin-Loaded Hydrogel

Curcumin-loaded hydrogels at 1.0% and 2.0% w/v were prepared as follows. Briefly, peptide stock solutions were prepared by dissolving lyophilized Fmoc-(3-Pal)_2_-NH_2_ in Milli-Q water to a final concentration of 30 mg mL^–1^. Separately, curcumin was dissolved in DMSO at a stock concentration of 40 mg mL^–1^. To obtain the final formulation, a small volume of the curcumin stock solution was added to the peptide solution, followed by the addition of an appropriate volume of double-distilled water to reach a final peptide concentration of 1.0% or 2.0% w/v, and a curcumin content of 0.05, 0.1, and 0.2% w/v. The mixture was sonicated for 30 s and incubated at room temperature until the formation of a uniform red self-supporting hydrogel.

### 2.11 Drug Loading Rate and Releasing Behaviors

The curcumin release from the hydrogels was investigated. To this purpose, 100 μL of 2.0% w/v hydrogels were prepared both in the presence and in the absence of curcumin. For drug-loaded samples, curcumin was incorporated at a final concentration of 2 mg mL^–1^ prior to gelation, yielding curcumin-loaded hydrogels used for release studies, while unloaded hydrogels were prepared under identical conditions and used as controls. After hydrogel formation, 1 mL of phosphate buffer (pH 7.4) or acetate buffer (pH 4.5) was added to each hydrogel sample, which was then incubated at room temperature. At each predetermined sampling time point (from day 0 to day 34), the release medium was carefully removed and replaced with 1 mL of fresh buffer, and this procedure was repeated until the final collection time point. The collected solutions were analyzed to quantify the amounts of released curcumin and dipeptide. Quantification was performed by HPLC, using the same column and gradients. Aliquots of the release media were directly injected into the HPLC system, and curcumin and dipeptide were identified based on their characteristic retention times. Quantitative analysis was carried out using calibration curves constructed from standard solutions of curcumin and dipeptide at known concentrations. The amount of each compound released at each time point was calculated from the corresponding peak areas. Incremental release values were calculated from the integrated peak areas obtained for each sampling time. Due to complete replacement of the release medium at every time point, cumulative release profiles were reconstructed by summing the incremental amounts released over time. All release experiments were performed in duplicate, and data are reported as mean values ±standard deviation, unless otherwise stated.

Release data were fitted by using the Korsmeyer–Peppas equation

$$\frac{Mt}{M\infty }=kt^{n}$$

here, *M*_*t*_ is the amount released at time *t*, *M*_∞_ is the amount released at infinite time, *k* represents the kinetic constant incorporating structural and geometric characteristics of the gel matrix (assumed as 0.01), and *n* is the release exponent, which indicates the release mechanism.

From day 0 to day 10, the residual hydrogel mass was also monitored and normalized to the initial mass to calculate the percentage of hydrogel mass retention, enabling the assessment of pH-dependent hydrogel stability over time.

## 3 Results and Discussion

### 3.1 Design, Synthesis, and Self-Assembly Characterization of Fmoc-(3-Pal)_2_-NH_2_

Classical Fmoc-dipeptides, particularly Fmoc-FF, despite their widespread use as low-molecular-weight hydrogelators, suffer from significant limitations, including poor aqueous solubility, which often necessitates the use of organic solvents such as DMSO to enable dissolution and trigger self-assembly.^35,36^ In addition, their gelation is typically restricted to acidic pH, making them unsuitable for in situ gelation under physiological conditions. This limitation is particularly critical given that these systems are often poorly injectable, further compromising their practical use in biomedical applications.^20^ To enlarge the plethora of gelating peptides, both phenylalanine residues were replaced with β-(3-pyridyl)-l-alanine, a heteroaromatic building block, whose pyridine ring (p*K*_a_ ≈ 5.3) increases polarity and potentially enables hydrogel formation under near-neutral pH conditions. Based on this strategy, the novel dipeptide Fmoc-(3-Pal)_2_-NH_2_ (Figure 1A) was developed and represents the focus of the present study. The dipeptide was synthesized via a standard solid-phase peptide coupling strategy (SPPS) and purified by preparative RP-HPLC chromatography. Structural identity and purity were confirmed by high-resolution mass spectrometry and ^1^HNMR and ^13^CNMR spectroscopy. The complete spectroscopic characterization is reported in the Supporting Information (Figures S1–S3).

**Figure 1:**
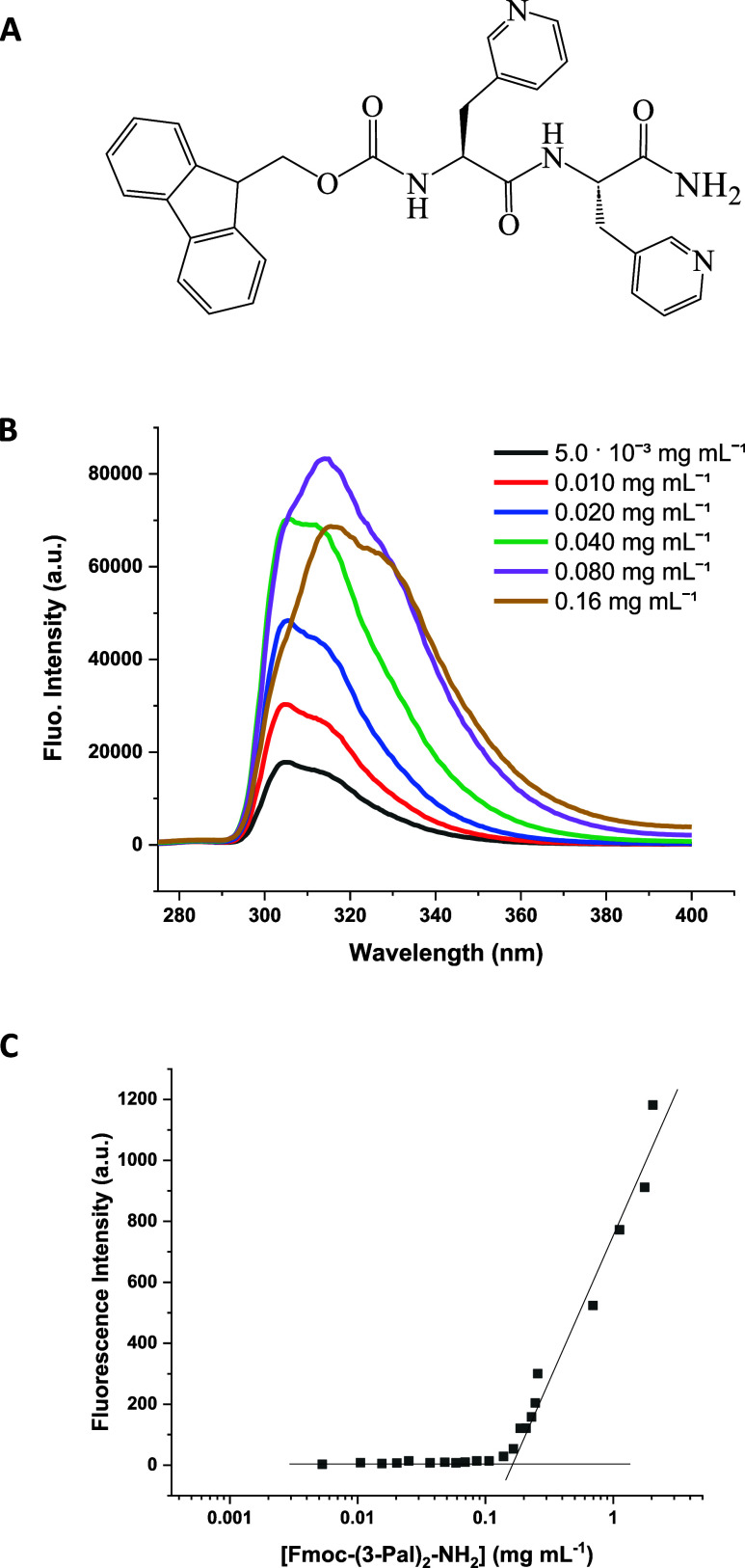
Structure of the Fmoc-(3-Pal)_2_-NH_2_. (B) The intrinsic fluorescence upon excitation at 260 nm of the peptide at concentrations between 5.0 × 10^–3^ and 0.16 mg mL^–1^ (C) CAC determination. Fluorescence intensity of the ANS fluorophore at 470 nm versus the concentration of the peptide. The CAC value was calculated from the break point.

The self-assembly behavior of Fmoc-(3-Pal)_2_-NH_2_ in phosphate-buffered saline (0.1 mol L^–1^ PBS, pH 8.0) was initially investigated through autofluorescence spectroscopy, exploiting the intrinsic fluorescence of the aromatic moieties upon excitation at 260 nm. This pH was selected to ensure that the pyridine ring remains unprotonated, with less than 1% present as the pyridinium ion. Protonation introduces a positive charge, which increases the peptide’s solubility in water but also promotes electrostatic repulsion between peptide chains, thereby hindering the formation of supramolecular aggregates. As shown in Figure 1B, the fluorescence emission intensity progressively increases with the concentration, reaching a maximum at 0.08 mg mL^–1^. Interestingly, at a higher concentration, the emission maximum exhibited a noticeable red shift accompanied by a decrease in intensity, suggesting the onset of a larger aggregate formation. The observed bathochromic shift and fluorescence quenching reflect enhanced molecular packing and restricted mobility of the aromatic moieties, pointing to the formation of ordered π–π-stacked domains characteristic of supramolecular nanostructures. These results confirm a concentration-dependent self-assembly, with a critical transition in the aggregation process occurring above 0.08 mg mL^–1^.

To further explore the kinetics of this self-assembly, time-dependent fluorescence experiments were performed. The evolution of the emission profile over 60 min, recorded at a peptide concentration of 0.16 mg mL^–1^, revealed a rapid initial decrease in the fluorescence intensity within the first 10 min, followed by a slower saturation phase that stabilized after approximately 30 min (Figure S4A). Notably, a progressive red shift of the emission maximum was also observed over time. This combination of intensity decay and spectral shift is again indicative of the progressive packing and structural reorganization of the peptide aggregates. A comparison with the fluorescence behavior at a higher peptide concentration (0.32 mg mL^–1^), shown in Figure S4B, highlights significant differences: at this concentration, the red-shifted emission maximum is already evident at time zero, suggesting that aggregation is more advanced from the outset. Moreover, the kinetics appear evidently faster, with the fluorescence signal reaching a stable plateau within the first 15 min.

The critical aggregation concentration (CAC) was then quantitatively estimated using a fluorescence titration with 8-anilinonaphthalene-1-sulfonic acid (ANS), a solvatochromic dye that exhibits increased fluorescence intensity in hydrophobic environments. In detail, ANS is a fluorophore that emits in the 460–480 nm range only when located in a hydrophobic environment, such as the dry interface of a nanostructure. Upon increasing the concentration of the peptide, the fluorescence emission of ANS exhibited a sharp increase starting around 0.10 mg mL^–1^, indicating the formation of hydrophobic domains typically associated with the early stages of supramolecular aggregation (Figure 1C). By plotting its fluorescence intensity as a function of peptide concentration, we can easily extrapolate the CAC value from the break points. The CAC was calculated to be approximately 0.17 mg mL^–1^, confirming the results of fluorescence experiments.

Finally, the secondary organization of the assembled structures was analyzed by optical dichroism (CD) spectroscopy. The CD spectra (Figure S4C) displayed two characteristic minima at approximately 215 and 200 nm. This pattern is indicative of a disordered conformation, with contributions from loosely packed β-sheet-like structures or early supramolecular aggregation. The presence of a shoulder at 215 nm suggests some degree of intermolecular organization, although no well-defined secondary structure is observed, as reported in other similar systems.^37,38^ The signal intensity and shape evolved with concentration, indicating a cooperative and ordered assembly process.

### 3.2 Formulation and Structural Properties of the Fmoc-(3-Pal)_2_-NH_2_ Hydrogel

To evaluate the gelation capability of Fmoc-(3-Pal)_2_-NH_2_, a range of concentrations (1, 2, 5, and 10 mg mL^–1^, corresponding to 0.1, 0.2, 0.5, and 1% w/v) was tested. Gel formation was assessed by using the vial inversion (tilt) method. The gelation tests were carried out by diluting the peptide stock solution (in water 30 mg mL^–1^) in a buffer solution at pH 8.0, a condition that promotes self-assembly through the complete deprotonation of the pyridyl moieties, enhancing thus intermolecular interactions. As shown in Figure 2A, at lower concentrations (0.1 and 0.2% w/v), the samples remained as clear solutions, whereas the formation of a hydrogel was observed at concentrations of 5.0 and 10.0 mg mL^–1^ (0.5 and 1.0% w/v, respectively). Moreover, the gelation tests were carried out in sodium phosphate solution at pH 6.0 to investigate the tunability of this system with respect to the pH. Since pH 6.0 is close to the p*K*_a_ of the para-substituted pyridyl groups, approximately 50% of these moieties are expected to be protonated at this pH. Under these conditions, hydrogel formation does not occur at 0.2% and 0.5% w/v, while successful gelation is observed at 1.0% w/v (Figure 2B); however, the resulting hydrogels appear more transparent compared to those formed at pH 8.0, thus suggesting differences in the network density or aggregate organization.

**Figure 2:**
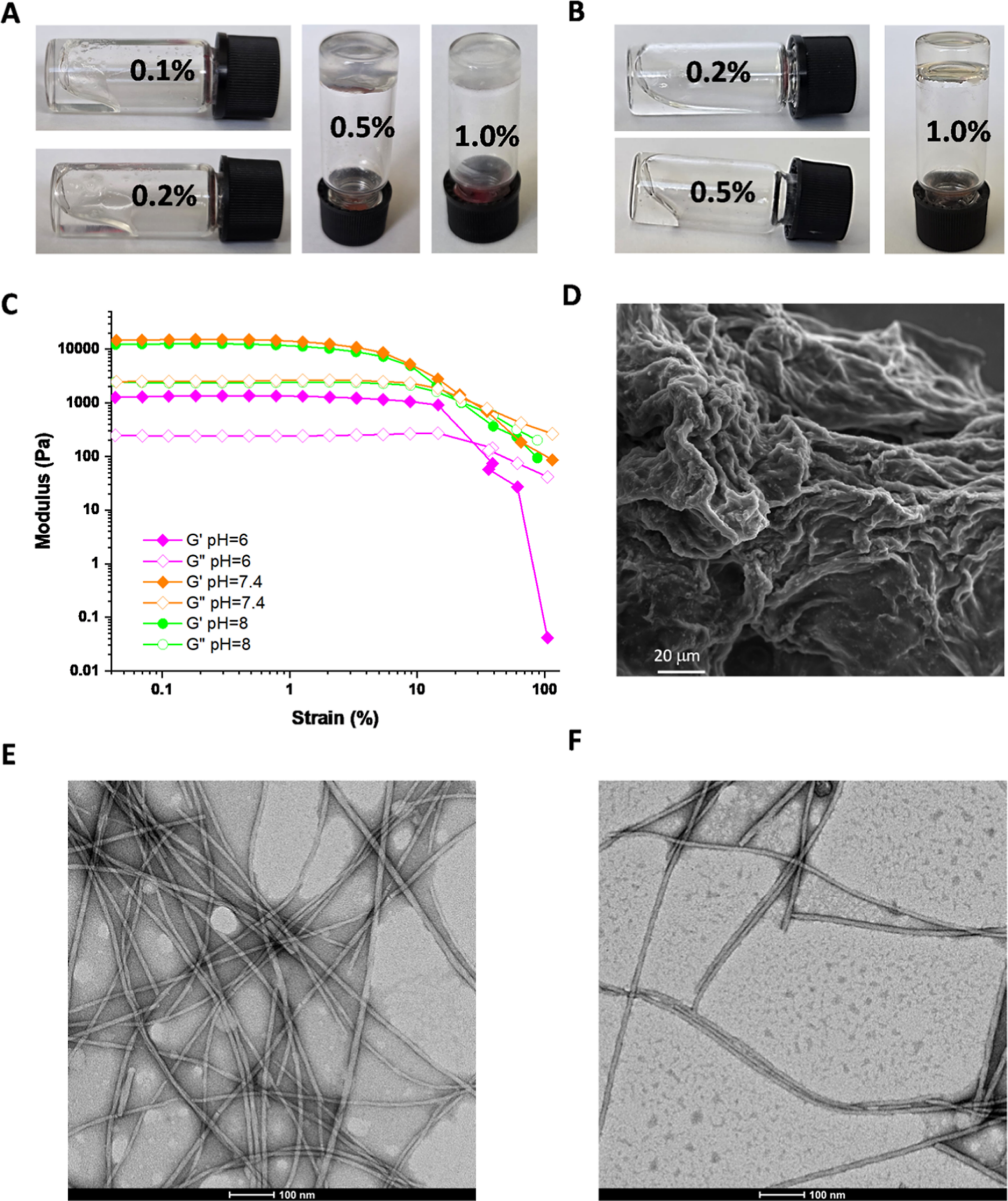
Three-dimensional (3D) hydrogel characterization. (A) Inverted tubes of the peptide solution at different concentrations (0.1, 0.2, 0.5, 1.0% w/v), in PBS at pH = 8.0. (B) Inverted tubes of the peptide solutions at different concentrations (0.2, 0.5, and 1.0% w/v), in acetate buffer at pH = 6.0. (C) Mechanical properties through strain sweep experiments of the peptide-based hydrogels formed with 1.0% w/v Fmoc-(3-Pal)_2_-NH_2_ at pH 6, pH 7.4, and 8. (D) SEM microphotos of 1.0% w/v Fmoc-(3-Pal)_2_-NH_2_ hydrogel, formed at pH = 8.0 (magnification 1800×, scale bar 20 μm). (E) TEM image of fibrillar assemblies at pH 6.0. (F) TEM image of fibrillar assemblies at pH 8.0 (scale bars: 100 nm).

The mechanical performance of the hydrogels was evaluated through oscillatory rheology (Figure 2C), focusing on the viscoelastic properties of 1.0% w/v hydrogels formed at pH 6.0 and pH = 8.0. In addition, the viscoelastic behavior of hydrogels formed at physiological pH (pH = 7.4) was also investigated for comparison. Strain sweep experiments were conducted to assess the linear viscoelastic region (LVR) and the structural robustness of the supramolecular network under increasing deformation. The resulting material formed at pH 8.0 (green line), exhibited a clear elastic response, with the storage modulus (*G*′) remaining consistently higher than the loss modulus (*G*″) throughout the LVR. This confirms the formation of a stable, solid-like hydrogel capable of withstanding mechanical stress without immediate structural failure. Remarkably, *G*′ values reached approximately 12 kPa, significantly higher than those typically reported for the reference Fmoc-FF hydrogel, which generally exhibits moduli in the range of 100–1000 Pa under similar conditions.^21^ This pronounced increase in mechanical strength is likely attributable to the specific contribution of the pyridyl rings, which not only support additional π–π stacking but also could engage in directional hydrogen bonding and probably enhance dipole–dipole interactions, contributing to a more tightly packed and cohesive network. The high modulus and extended LVR suggest that the hydrogel retains its structural integrity even under moderate deformation, making it particularly attractive for applications requiring mechanical elasticity such as injectable drug depots. Hydrogels formed at pH 7.4 (orange line) displayed viscoelastic properties very similar to those observed at pH 8.0, with values in the same order of magnitude. The comparable mechanical performance observed at pH 7.4 and 8.0 can be rationalized by the nearly complete deprotonation of the pyridyl groups under both conditions (≈99.2% at pH 7.4 and ≈99.8% at pH 8.0, calculated using the Henderson–Hasselbalch equation; see the Materials and Methods), which promotes analogous supramolecular interactions and network packing efficiency. A concise summary of the deprotonation state of the pyridyl group, the storage modulus, the linear viscoelastic range, the loss factor, and the corresponding gelation efficiency at the different pH values is reported in Table 1.

**Table 1:** Summary of Pyridyl Deprotonation, Plateau Storage Modulus (*G*′), Linear Viscoelastic Range, Loss Factor (tan δ), and Gelation Efficiency of 1.0% w/v Hydrogels at Different pH Values

pH	% deprotonated pyridyl	*G*′ in LVR (Pa)	LVR strain range (%)	tan δ = *G*″/*G*′	macroscopic gelation efficiency
6.0	≈87.0%	∼1200 Pa	0.1–1%	0.18 ± 0.01	weak/soft gel
7.4	≈99.2%	∼13,000 Pa	0.1–10%	0.18 ± 0.01	strong gel
8.0	≈99.8%	∼12,500 Pa	0.1–12%	0.19 ± 0.01	strong gel

The self-healing properties of the hydrogel (1.0% w/v, pH 8) were investigated by step-strain rheological measurements at a constant frequency (ν = 1.0 Hz). The material was subjected to alternating low strain (γ = 0.1%, 60 s), within the linear viscoelastic region, and high strain (γ = 80%, 30 s) for two consecutive cycles. As shown in Figure S5, the application of high strain led to a significant decrease in the storage modulus, indicating the disruption of the hydrogel network. Notably, upon restoring the strain to low values, *G*′ rapidly recovered to its initial value, demonstrating the ability of the system to reconstruct its internal structure. This reversible recovery of viscoelastic properties confirms the self-healing behavior of the hydrogel.

Under mildly acidic conditions (magenta line), the hydrogel exhibited a markedly softer viscoelastic profile, with *G*′ values around 1000 Pa and a significantly narrower LVR. This reduction in stiffness can be attributed under these conditions to approximately 15% protonation of the pyridyl nitrogen atoms, which weakens π–π stacking and hydrogen bonding, thereby disrupting the efficiency of the self-assembly process (Table 1). The network formed at pH 6.0 is thus less densely packed and more prone to deformation, suggesting that the gelation process is highly sensitive to the ionization state of the pyridyl group. Therefore, materials with tunable mechanical properties can be obtained simply by adjusting the pH, allowing adaptation to different application requirements.

Scanning Electron Microscopy provided insights into the morphology of 1.0% w/v hydrogel formed at pH 8.0 (Figure 2D), revealing an interconnected porous matrix composed of entangled fibrillar bundles. This nanofibrous architecture is typical of supramolecular peptide assemblies, playing a critical role in entrapping water and potentially enabling diffusion-controlled release. To characterize the organization of peptide moieties into the hydrogel network, Fourier Transform Infrared (FT-IR) spectroscopy was performed in the amide I region (1600–1700 cm^–1^). The spectrum (Figure S6) displayed a broad absorption band centered at approximately 1645 cm^–1^, indicative of β-sheet-like hydrogen bonding commonly observed in peptide-based hydrogels. This suggests that the self-assembled fibrillar network is stabilized by extensive intermolecular H-bonding interactions.

To further probe the nanoscale organization of the assemblies, TEM analyses were performed at low concentrations (0.1% w/v) under both pH 6.0 and pH 8.0 conditions (Figure 2E,F, respectively). The samples displayed entangled fibrillar networks with fibril diameters in the 12–14 nm range and lengths extending over several micrometers. No substantial differences in the nanoscale morphology were observed between the two pH values, indicating that the fundamental fibrillar architecture is preserved. These observations support the conclusion that pH probably modulates higher order organization and macroscopic gel properties, while having a limited effect on the fibrillar structure under these conditions.

### 3.3 Hydrogel Stability and Biocompatibility

The hydrogel’s stability was further investigated under two different pH conditions to assess its responsiveness to physiological and acidic environments (Figure 3A). Stability tests were performed by immersing the hydrogels in buffer solutions at pH 7.4 and 4.5, respectively, and measuring the residual mass each day over a period of 10 days. At pH 7.4, the hydrogel remained stable with minimal degradation (∼15%) after 10 days, suggesting good solvation resistance under physiological conditions. In contrast, at pH 4.5, the hydrogel progressively fragmented over time, likely due to protonation of the pyridyl nitrogen atoms, which weakens intermolecular interactions and destabilizes the network. After 10 days, 50% of the initial hydrogel mass was lost, confirming significant degradation under acidic conditions.

**Figure 3:**
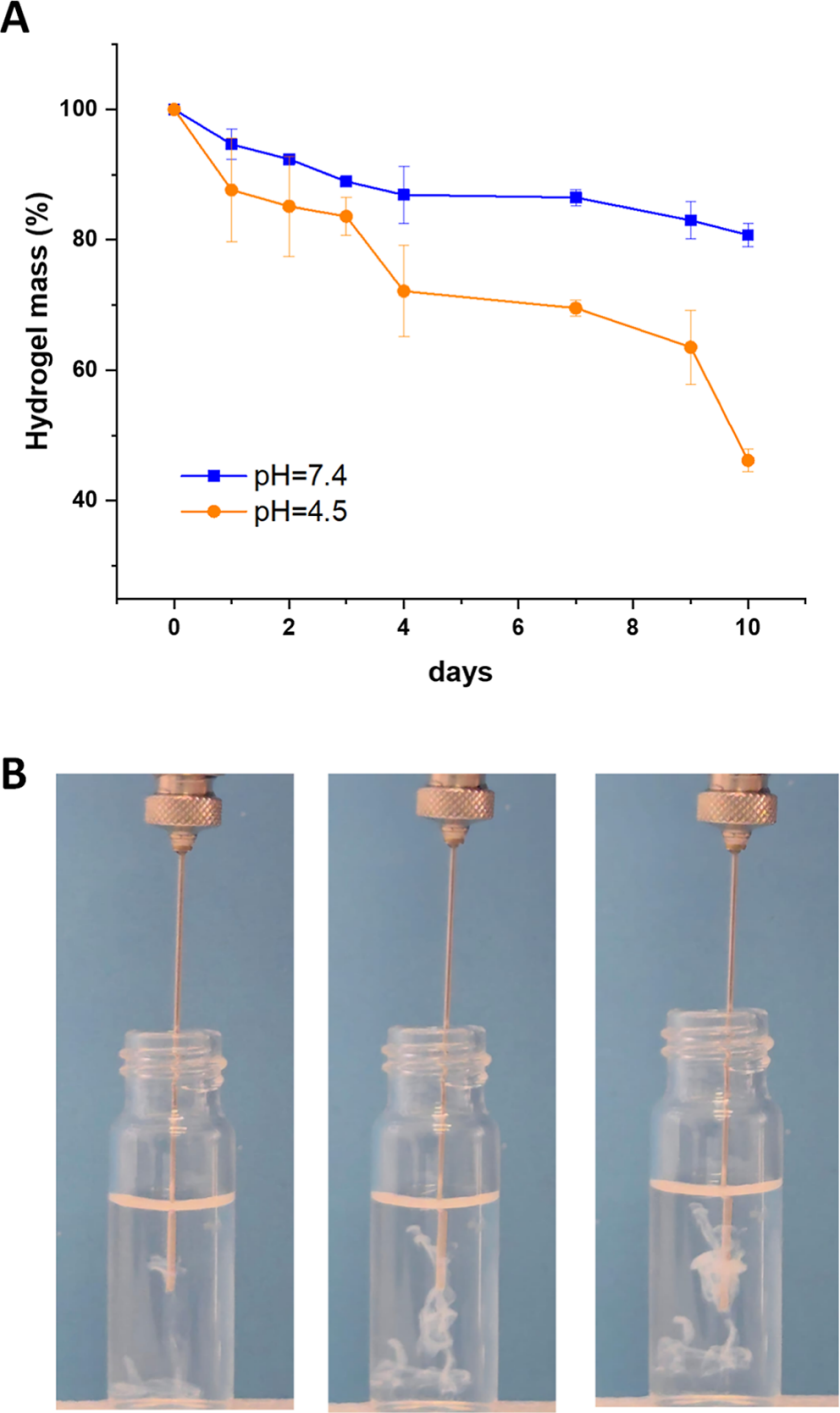
(A) Degradation percentage of 1.0% w/v hydrogel over time under two different pH conditions. (B) In situ hydrogelation studies of Fmoc-(3-Pal)_2_-NH_2_. Photographs represent the sequence of events involved in in situ gelation and the appearance of the formed Fmoc-(3-Pal)_2_-NH_2_ hydrogel upon injection into phosphate solution (0.1 mol L^–1^, pH 7.4).

The ability of Fmoc-(3-Pal)_2_-NH_2_ to form in situ hydrogels was assessed by injecting peptide solutions directly into phosphate-buffered saline (0.1 mol L^–1^, pH 7.4) at room temperature. Aqueous stock solutions of the peptide at 10 mg mL^–1^ were loaded into a standard laboratory syringe. Upon manual injection into buffer solution, instantaneous gelation was observed, as confirmed by images in Figure 3B. The transition from sol to gel occurred without the need for organic solvents, heating, or external cross-linker agents. The viscoelastic properties of the resulting in situ formed hydrogel were further characterized by oscillatory rheology, revealing *G*′ values comparable to those measured for hydrogels prepared by conventional mixing, thereby confirming that in situ gelation does not compromise the mechanical integrity of the network (Figure S7).

Unlike the widely studied Fmoc-FF-based hydrogels, which typically require acidic conditions or the use of organic cosolvents to trigger gelation,^39^ Fmoc-(3-Pal)_2_-NH_2_ appears to be a promising system for injectable applications, as it allows hydrogel formation under mild and biocompatible conditions.

In order to further assess its suitability for biomedical applications, the in vitro cytotoxicity of Fmoc-(3-Pal)_2_-NH_2_ hydrogels was evaluated using HEK-293 cells. Cells were incubated for 48 h at 37 °C with conditioned media obtained after 16 h incubation of the hydrogels at 1 and 0.5% w/v. Cell viability was determined by an MTT assay. As shown in Figure S8, no significant cytotoxicity was observed. Cell viability remained high, with values of approximately 93% and 85% for hydrogels at 1 and 0.5% w/v, respectively. These results confirm the good biocompatibility of the system and support its potential for biomedical applications.

### 3.4 Encapsulation of Curcumin and Hydrogel Properties

To explore the applicability of the Fmoc-(3-Pal)_2_-NH_2_ hydrogel as drug delivery platforms, we evaluated its ability to encapsulate curcumin, a poorly water-soluble polyphenolic compound with known anti-inflammatory and anticancer properties. Compared to free curcumin, the sustained release achieved through dipeptide hydrogels may allow for lower drug dosages while enhancing therapeutic efficacy and promoting prolonged biological activity.^40,41^ To investigate the properties of drug-loaded hydrogels, the 1.0% w/v curcumin-loaded Fmoc-(3-Pal)_2_-NH_2_ hydrogels at different drug concentrations (curcumin at 0.05, 0.1, and 0.2% w/v) were successfully prepared under physiological pH (Figure 4A). All the curcumin-loaded hydrogels are homogeneous and not present any syneresis related to the drug encapsulation. For this reason, we performed the further studies only for the hydrogel containing the highest curcumin concentration (0.2% w/v). Besides, the mechanical properties of this hydrogel were also characterized (Figure 4B). The results (magenta line) showed that the storage modulus *G*′ of curcumin-loaded hydrogels slightly decreased compared with that of the blank hydrogels (Figure 2C, green line), suggesting that the encapsulated curcumin had only minimal effects on the mechanical properties of hydrogels, with a slight decrease in values from 12.5 to 11.3 kPa. Moreover, a slight shift in the critical strain at which the network starts to break down was observed: while the blank hydrogel maintained its elastic behavior up to approximately 10% strain, the curcumin-loaded hydrogel exhibited a reduction in *G*′ starting at around 8% strain, suggesting a modest weakening of the network’s mechanical stability upon drug loading. Although a self-supporting hydrogel was macroscopically formed, these observations suggest that curcumin could interfere with the hydrophobic and π–π stacking interactions essential for peptide self-assembly. Given the large aromatic surface area of curcumin, it is plausible that it either competes for stacking with the fluorenyl and pyridyl units or inserts itself between assembling peptides, thereby destabilizing the network. To counteract this slight destabilization and recover the mechanical integrity of the gel, we increased the peptide concentration to identify the optimal ratio for encapsulating the same amount of curcumin. Specifically, we investigated Fmoc-(3-Pal)_2_-NH_2_ at a concentration of 2.0% w/v (20 mg mL^–1^), while keeping the curcumin content constant (0.2% w/v). At this concentration, a robust hydrogel was obtained, as confirmed by the vial inversion test (Figure S9A). Rheological characterization of the 2.0% curcumin-loaded hydrogel (Figure 4B, blue line) confirmed a substantial enhancement in mechanical strength, with a storage modulus (*G*′) that not only exceeded the unloaded system (approximately 23.0 kPa, data not shown) but reached an extraordinarily high value of approximately 30.0 kPa. This is a notable result for a dipeptide-based supramolecular hydrogel, and suggests that the presence of curcumin at higher peptide concentrations may contribute to the densification and reinforcement of the supramolecular network, possibly by stabilizing stacking domains once sufficient peptide is present to support network formation.^41^ The mechanical properties of hydrogels result comparable to many soft tissues and consistent with previously reported peptide hybrid hydrogels.^41,42^

**Figure 4:**
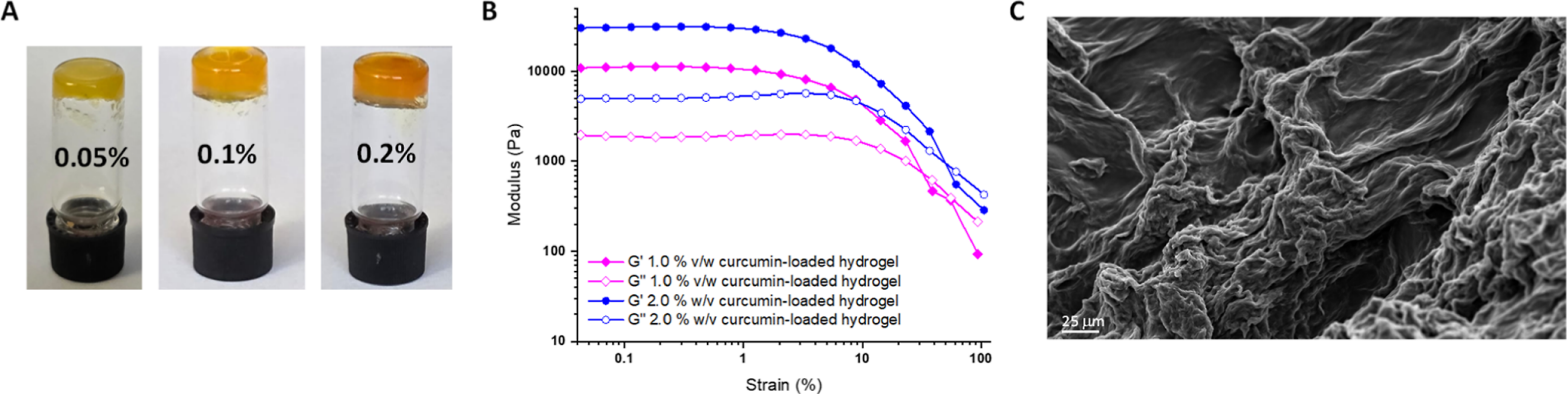
Three-dimensional curcumin-loaded hydrogel characterization. (A) Inverted test tubes of the peptide at a concentration 1% w/v in 0.1 mol L^–1^ PBS at pH 8.0, in the presence of 0.05, 0.1, 0.2% w/v of curcumin. (B) Mechanical properties through strain sweep experiments of the peptide-based hydrogels formed with 1.0 and 2.0% w/v Fmoc-(3-Pal)_2_-NH_2_ loaded with 0.2% of curcumin. (C) SEM microphotos of 2.0% w/v curcumin-loaded hydrogel.

The hydrogel also exhibited remarkable thermal stability, as it retained its gel state even after heating. The thermal stability was quantitatively assessed through a temperature sweep experiment, in which the *G*′ and *G*″ moduli were monitored upon heating from 25 to 80 °C. As shown in Figure S9B, the hydrogel retained a solid-like behavior throughout the entire temperature range, with *G*′ remaining consistently higher than *G*″, indicating preservation of the gel state without transition to a viscous solution.

The microstructure of the 2.0% curcumin-loaded hydrogel was examined by SEM (Figure 4C), revealing a dense, multilayered, and fibrillar network morphology, similar to the unloaded system. This indicates that the peptide is capable of maintaining effective supramolecular interactions even in the presence of a hydrophobic cargo, provided that the concentration exceeds a threshold necessary to support network formation.

### 3.5 Stability and Release Properties of Curcumin-Loaded Hydrogels

The drug loading capacity and injectability of the hydrogels were also evaluated. The absence of syneresis in curcumin-loaded hydrogels suggests efficient incorporation of the drug into the supramolecular network. To further validate this assumption, 50 μL of the curcumin-loaded hydrogel was washed with 500 μL of PBS (pH 7.4), and analysis of the collected supernatant revealed no detectable amount of unencapsulated curcumin, confirming effective drug retention within the hydrogel matrix. The 2.0% curcumin-loaded hydrogels exhibited efficient in situ gelation, as demonstrated in Figure 5A, where the hydrogel rapidly formed upon injection into aqueous medium, indicating again its suitability for injectable applications.

**Figure 5:**
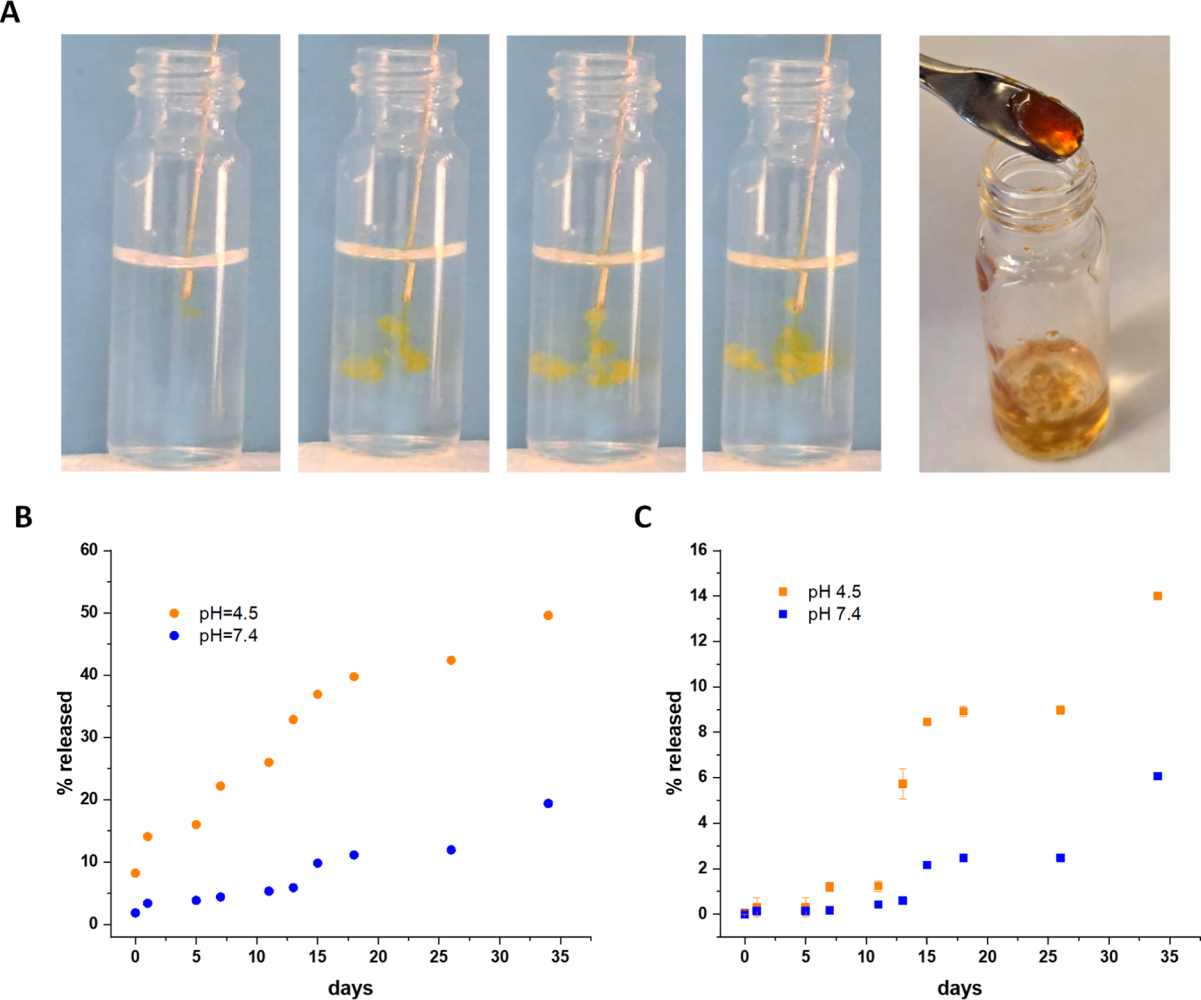
(A) In situ hydrogelation studies of 2.0% w/v Fmoc-(3-Pal)_2_-NH_2_ and 0.2% w/v of curcumin. Photographs represent the sequence of events involved in in situ gelation and the appearance of the formed curcumin-loaded hydrogel upon injection into 0.1 mol L^–1^ PBS (pH 8). Reconstructed cumulative release of dipeptide (B) and curcumin (C) after incubation in buffer at pH 4.5 and 7.4. Cumulative values were obtained by summing the amount released at each time point, as the release medium was completely replaced at every sampling.

In addition, the stability of both blank and curcumin-loaded hydrogels was monitored over 10 days at pH 4.5 and 7.4 by measuring the residual hydrogel mass (Figure S10). The results show that increasing the concentration of Fmoc-(3-Pal)_2_-NH_2_ significantly enhances the stability under acidic conditions. Hydrogels prepared at 2% (w/v) retained more than 90% of their mass after 10 days, whereas those at 1% (w/v) lost nearly 50%, as shown in Figure 3A.

On the basis of these stability results, the quantitative release behavior of curcumin and the dipeptide from the hydrogels was subsequently investigated under the same pH conditions.

As shown in Figure 5B, the dipeptide exhibits a slightly discontinuous release profile with alternating phases of limited release and more pronounced release events. This behavior is likely associated with gradual, time-dependent changes in the hydrogel structure or local physicochemical environment, rather than a strictly diffusion-controlled process. A marked pH dependence is observed, with generally enhanced release at pH 4.5 compared to pH 7.4, in agreement with the reduced hydrogel stability previously observed under acidic conditions.

The release behavior of curcumin is reported in Figure 5C. Similar to the case for the dipeptide, curcumin release proceeds through discrete release events rather than a gradual diffusion-controlled process, confirming that release is closely associated with changes in the hydrogel structure over time. Notably, curcumin release is significantly enhanced at pH 4.5, consistent with the partial destabilization of the hydrogel observed under acidic conditions. However, the overall amount of curcumin released results lower than that of the dipeptide, which may be influenced by its low hydrophilicity and by partial adsorption onto the hydrogel support and experimental surfaces (e.g., Petri dishes and microcentrifuge tubes), particularly given the small amounts involved. Curcumin release data were fitted using Korsmeyer–Peppas model, obtaining release exponents of approximately n = 0.91 (pH = 4.5) and n = 1.03 (pH = 7.4). This analysis, excluding a pure diffusion profile (Case I) and diffusion-controlled release, suggests a not purely Fickian behavior, more consistent with a matrix relaxation/swelling-controlled release (Case II), especially at pH 7.4.^43^

Overall, these findings demonstrate the tunable nature of the system, where the concentration of the gelator dictates hydrogel stability over time and thereby modulates the drug release profile. Finally, the release kinetics closely mirrored the degradation profile of the hydrogel, suggesting that curcumin is primarily retained within the supramolecular network and is gradually released as the hydrogel matrix disassembles. This behavior supports a degradation-driven release mechanism in which the breakdown of the fibrillar network enables the diffusion of the encapsulated hydrophobic molecules. The absence of a significant burst release further indicates strong interactions between curcumin and the peptide matrix, likely mediated by hydrophobic and π–π stacking interactions. These findings highlight the ability of the system to provide sustained and controlled release, making it a promising platform for the delivery of poorly soluble therapeutic agents.

## 4 Conclusions

In recent decades, short and ultrashort peptides have been considered as building blocks for the formulation of biocompatible materials, including hydrogels. Most studies have focused on systems containing phenylalanine and other aromatic natural residues,^44^ where the peptide sequence plays a key role in determining structural organization, mechanical properties, and water content of the resulting materials. These features are crucial for payload loading and controlled release, enabling applications in areas such as cancer therapy and antimicrobial treatments. In this work, we report a novel pH-responsive hydrogel based on the ultrashort dipeptide Fmoc-(3-Pal)_2_-NH_2_, in which the incorporation of a pyridine moiety introduces intrinsic protonation–deprotonation responsiveness. This design enables hydrogel formation under mild and near-physiological conditions, while allowing fine-tuning of mechanical properties as a function of pH. In particular, softer materials are obtained at pH 6, whereas stronger and more stable hydrogels are formed at pH 8, remaining stable for more than 10 days. In addition, rheological studies demonstrated enhanced mechanical performance compared with the widely studied Fmoc-Phe-Phe system, with higher storage modulus values and good structural recovery under an applied strain, confirming the formation of a robust and self-healing network. In contrast, under slightly acidic conditions (pH below 5.0), the hydrogel progressively disintegrated over time, enabling the release of payloads. This pH-dependent degradability is particularly advantageous, as it allows the modulation of the drug release profile based on the pH of the surrounding tissue, allowing selective and responsive delivery in different physiological or pathological environments. Moreover, the in situ gelation behavior, combined with the mild preparation conditions, highlights the potential of this system for injectable delivery applications. Importantly, the proposed system combines structural simplicity with functional responsiveness, as pH-tunable behavior is achieved within a single-component ultrashort dipeptide without the need for additional components or complex formulations. This distinguishes it from previously reported pH-responsive systems, which often rely on longer peptide sequences^45–48^ or multicomponent assemblies.^29,30^ As a proof of concept, curcumin was successfully loaded into the hydrogel without compromising its supramolecular organization. Notably, the release of the payload is sustained over longer time scales under mildly acidic conditions. Preliminary in vitro studies demonstrated good biocompatibility of the system, with high cell viability (>85%) observed after 48 h incubation, supporting its potential for biomedical applications.

Although this study focuses on the meta-substituted pyridine derivative, a systematic comparison with other pyridine isomers (e.g., 2- and 4-substituted analogues) could provide further insights into the role of positional isomerism on gelation behavior and will be the subject of future investigations.

Overall, these findings highlight the potential of Fmoc-(3-Pal)_2_-NH_2_ hydrogels not only for wound healing applications but also for broader in vivo biomedical applications.

## Supplementary Material



## Notes

ESI mass spectrum, ^1^H NMR, and ^13^C NMR spectra (PDF)

## Abbreviations

Fmoc: fluorenylmethyloxycarbonyl.
DIEA: N diisopropylethylamine.
HBTU: O-(benzotriazol-1-yl)-N,N,N′,N′-tetramethyluronium hexafluorophosphate.
HOBt: 1-hydroxybenzotriazole.
Pal: beta­(3-pyridyl)-l-alanine
CAC: critical aggregation concentration
