# Supplementary material for: Mechanically Enhanced Ultrashort Peptide Hydrogels for pH-Triggered Release
Source: ACS Polym Au. 2026 Apr 16;6(3):906–17. doi: 10.1021/acspolymersau.6c00020 (PMC13261726; doi:10.1021/acspolymersau.6c00020)
Supplement: Supplementary file 1 [file lg6c00020_si_001.pdf]

Supplementary Information

Mechanically Enhanced Ultrashort Peptide Hydrogels for pH-triggered release

Pasqualina Liana Scognamiglio[1,3] Carlo Diaferia[2,3], Mariantonietta Pizzella[4], Antonella Accardo[2,3], Giancarlo Morelli [2,3] and Diego Tesauro[2,3].

[1] Basic and Applied Sciences Department. University of Basilicata, – Potenza (Italy);

[2] Pharmacy Department. University of Naples “Federico II”,80131 – Napoli (Italy);

[3] Interuniversity Research Centre on Bioactive Peptides (CIRPeB) University of Naples “Federico II”, 80134 – Napoli (Italy);

[4] IRCCS SYNLAB SDN, Via Ferraris 144, 80146 Naples, Italy.

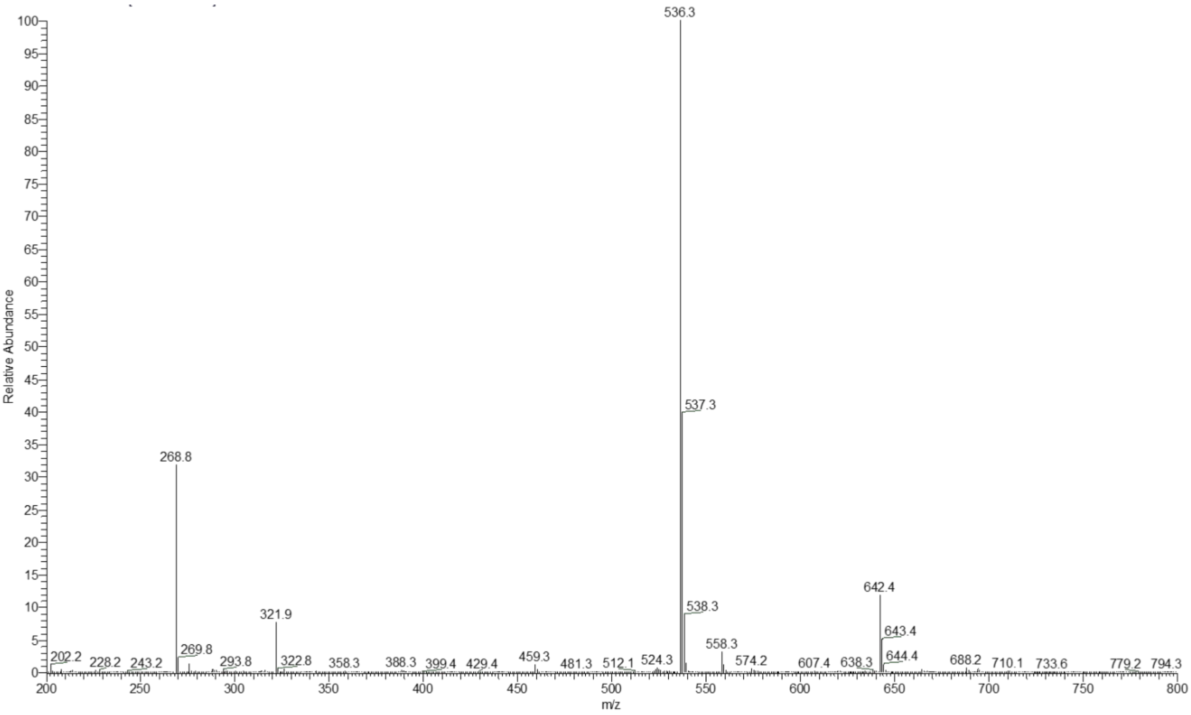

Figure S1. Mass spectrum of the analyzed compound, recorded in ESI mode. The molecular ion peak at *m/z* 536.3 corresponds to the $[M+H]^+$ species, consistent with the calculated molecular mass (calculated: 535.2 Da). A characteristic fragment ion is also observed at *m/z* 268.8, corresponding to the $[M+2H]^{2+}$ species.

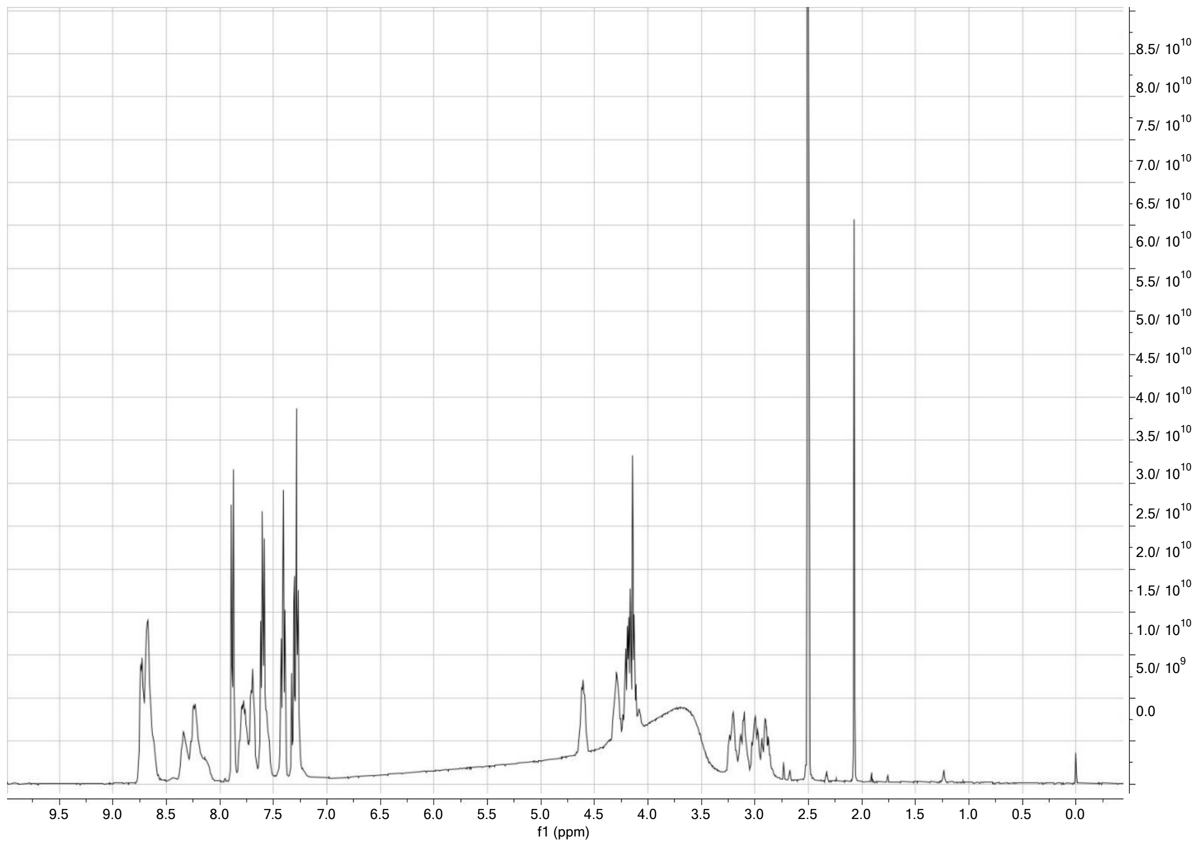

f1 (ppm)

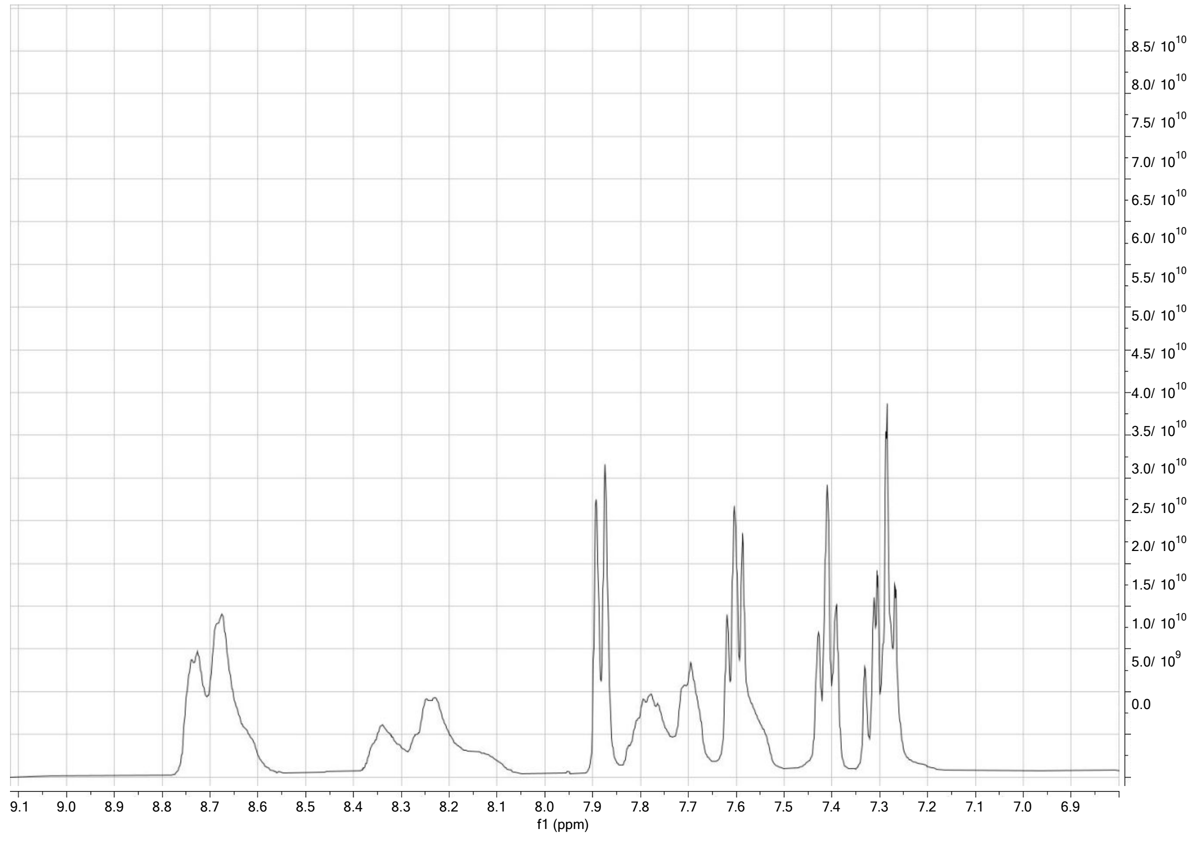

f1 (ppm)

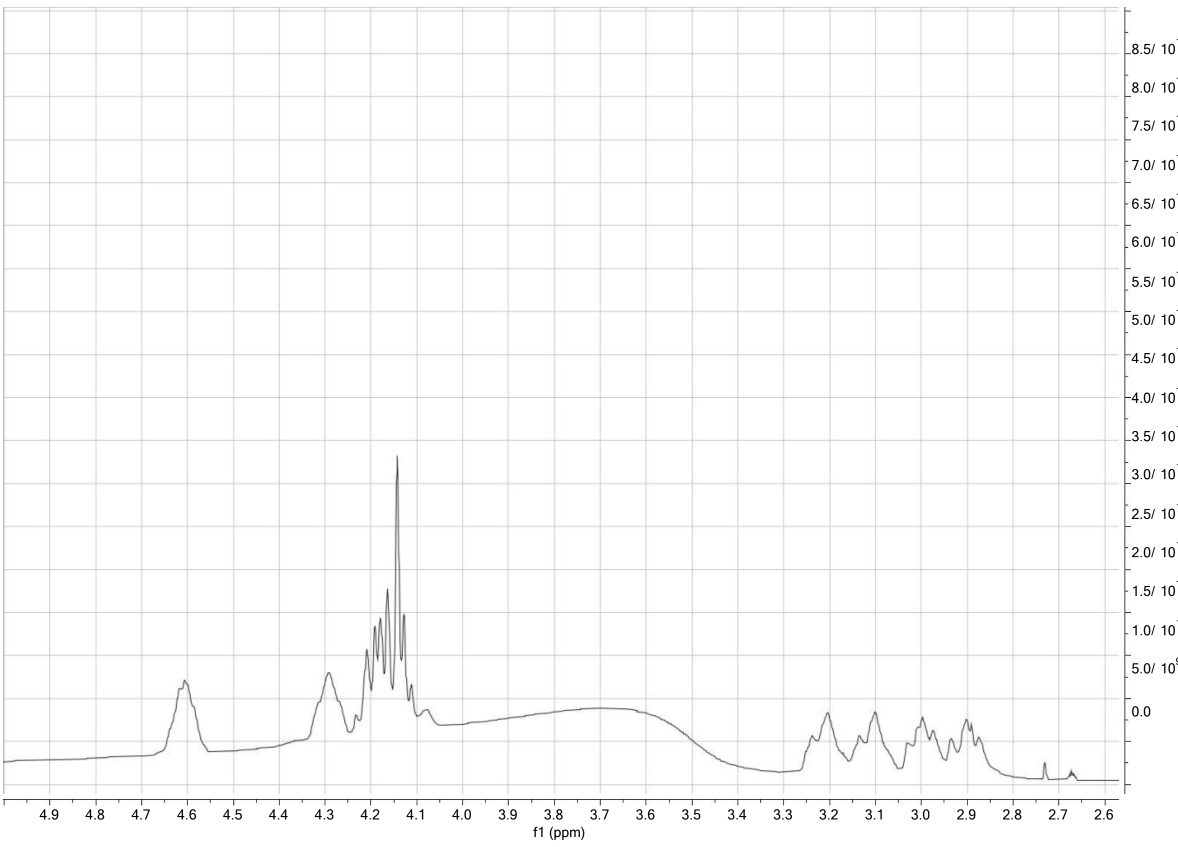

Figure S2. ^{1}H NMR spectrum of the analyzed compound, recorded in DMSO-d_6 at frequency, 400 MHz. The chemical shifts (δ, ppm) are consistent with the expected structure.

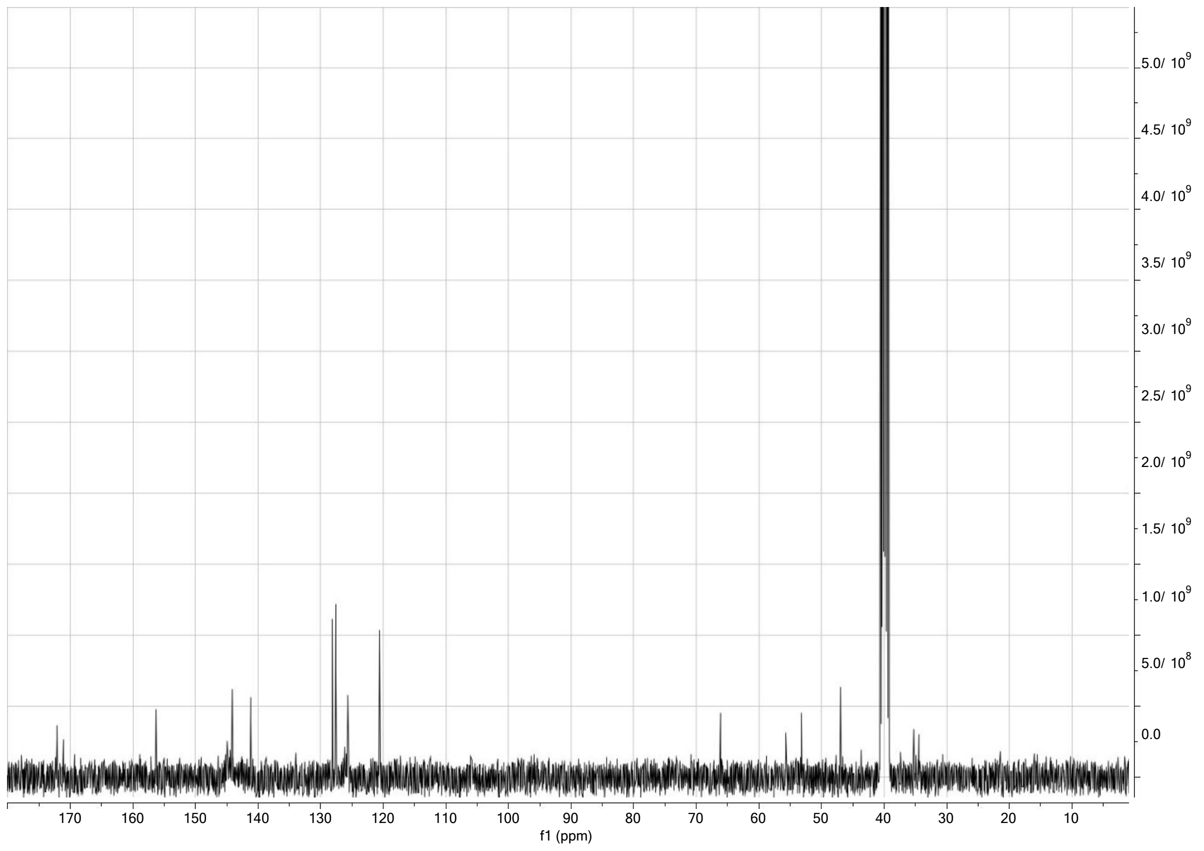

5.0/ 10^9
4.5/ 10^9
4.0/ 10^9
3.5/ 10^9
3.0/ 10^9
2.5/ 10^9
2.0/ 10^9
1.5/ 10^9
1.0/ 10^9
5.0/ 10^8
0.0
170 160 150 140 130 120 110 100 90 80 70 60 50 40 30 20 10
f1 (ppm)

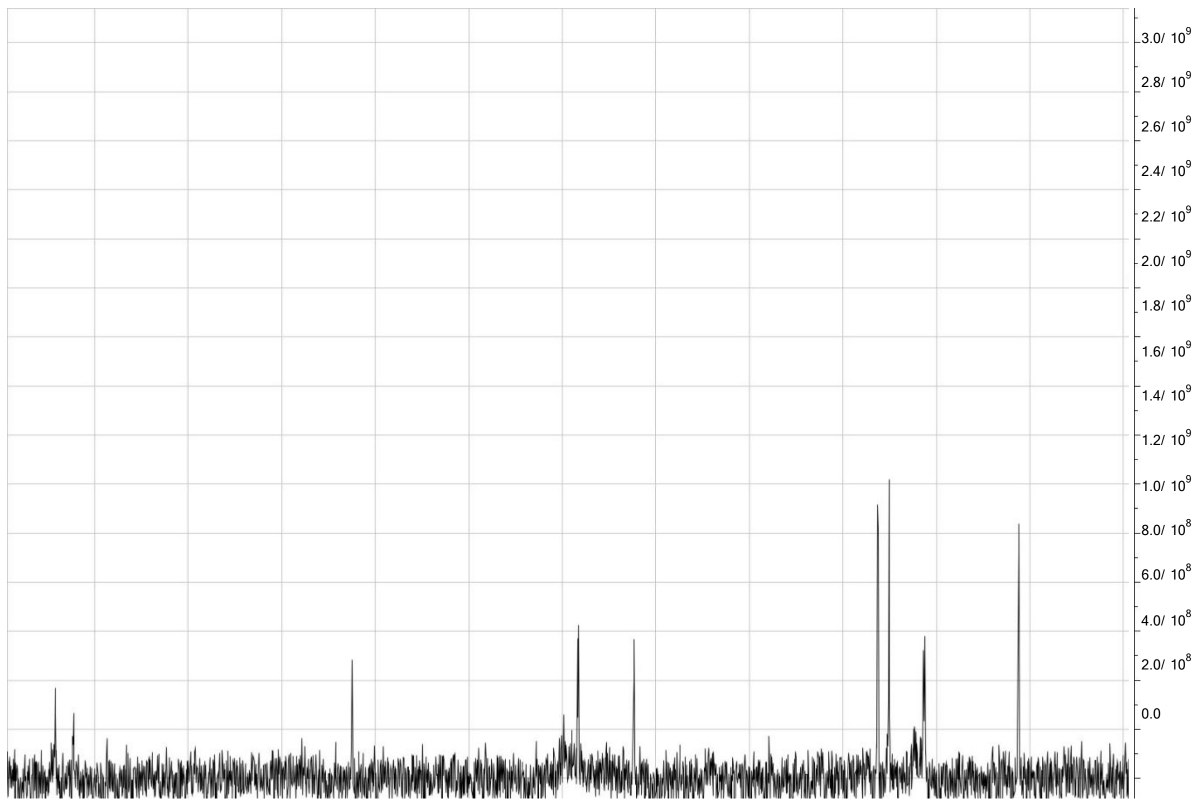

3.0/ 10^9
2.8/ 10^9
2.6/ 10^9
2.4/ 10^9
2.2/ 10^9
2.0/ 10^9
1.8/ 10^9
1.6/ 10^9
1.4/ 10^9
1.2/ 10^9
1.0/ 10^9
8.0/ 10^8
6.0/ 10^8
4.0/ 10^8
2.0/ 10^8
0.0
170 165 160 155 150 145 140 135 130 125 120 115
f1 (ppm)

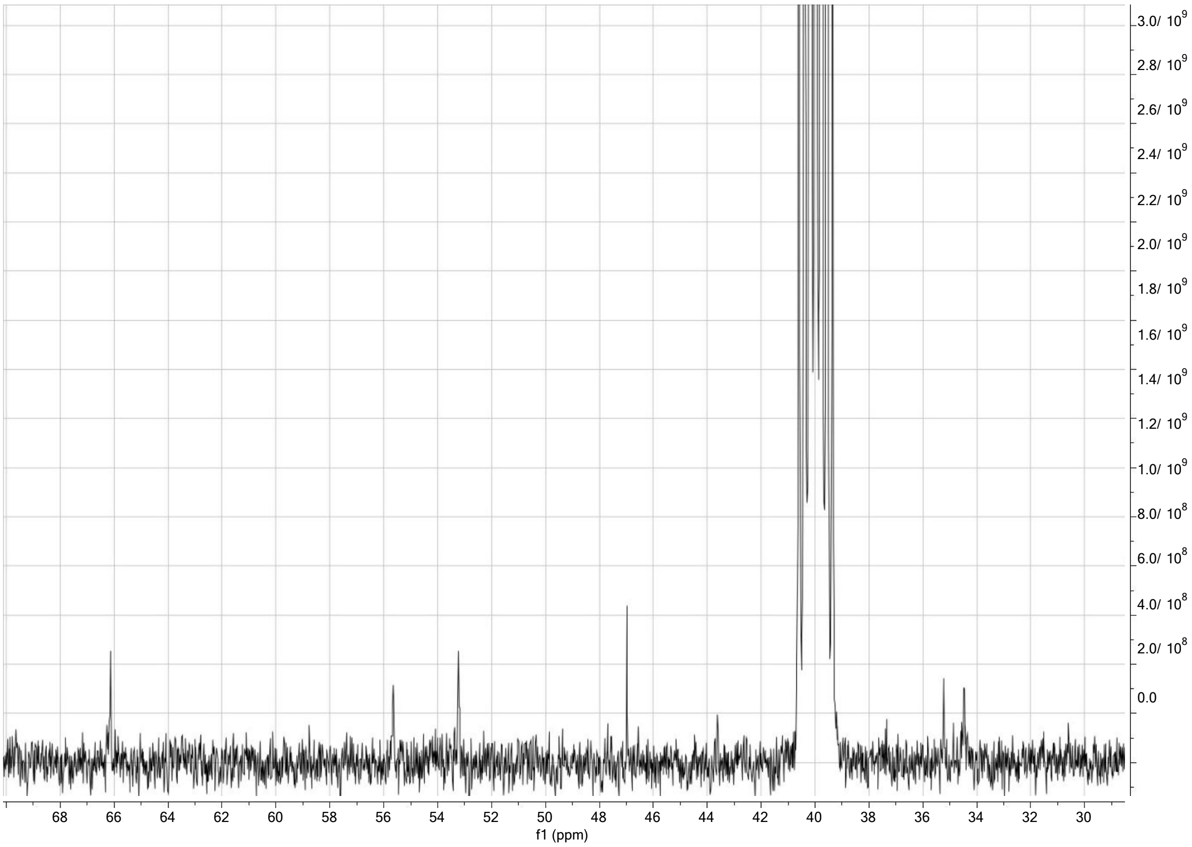

Figure S3. ^{13}C NMR spectrum of the analyzed compound, recorded in DMSO-d_6 at frequency 100 MHz. The observed resonances are in agreement with the proposed molecular framework.

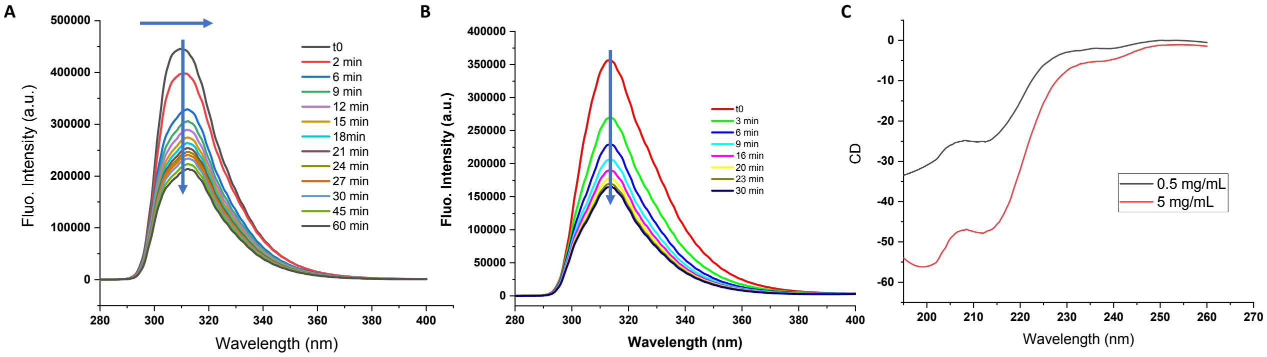

Figure S4. Time-dependent fluorescence and circular dichroism studies of the Fmoc-(3-Pal)$_2$-amide self-assembly. **(A)** Evolution of the fluorescence emission spectra (λ_{ex} = 260 nm) over 60 min at a peptide concentration of 0.16 mg mL^{-1}. **(B)** Fluorescence emission spectra recorded over 30 min at a peptide concentration of 0.32 mg mL^{-1}. **(C)** Circular dichroism spectra at 0.5 and 5 mg mL^{-1}.

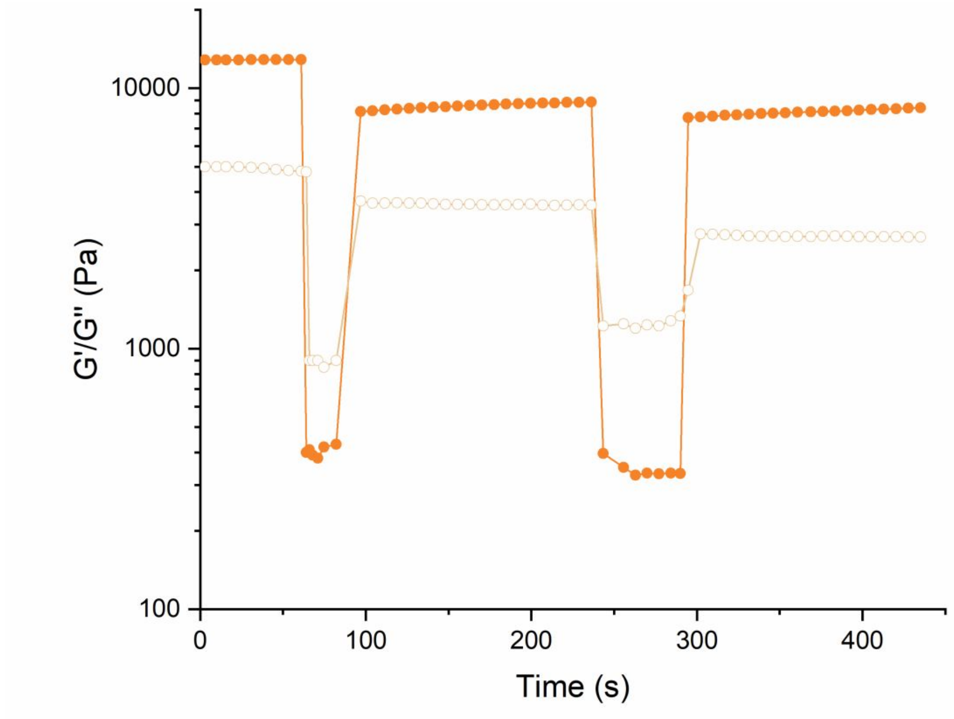

Figure S5. Step strain measurements for self-healing properties. G' (●) and G'' (○)

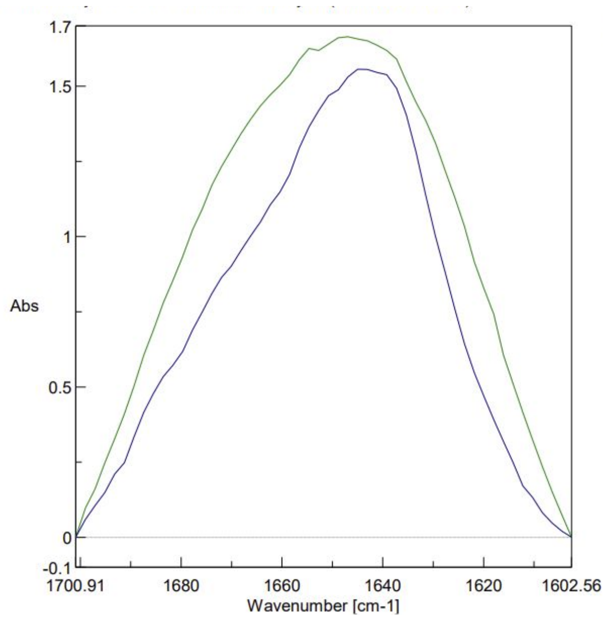

Alpha-Helix	Beta-Sheet	Beta-turn	Other
9%	31%	23%	25%

Figure S6. FT-IR spectrum of Fmoc-(3-Pal)$_2$-amide hydrogel in the amide I region (1600–1700 cm^{-1}). Secondary structure deconvolution analysis revealed contributions from α-helix (9%), β-sheet (31%), β-turn (23%), and random/other structures (25%).

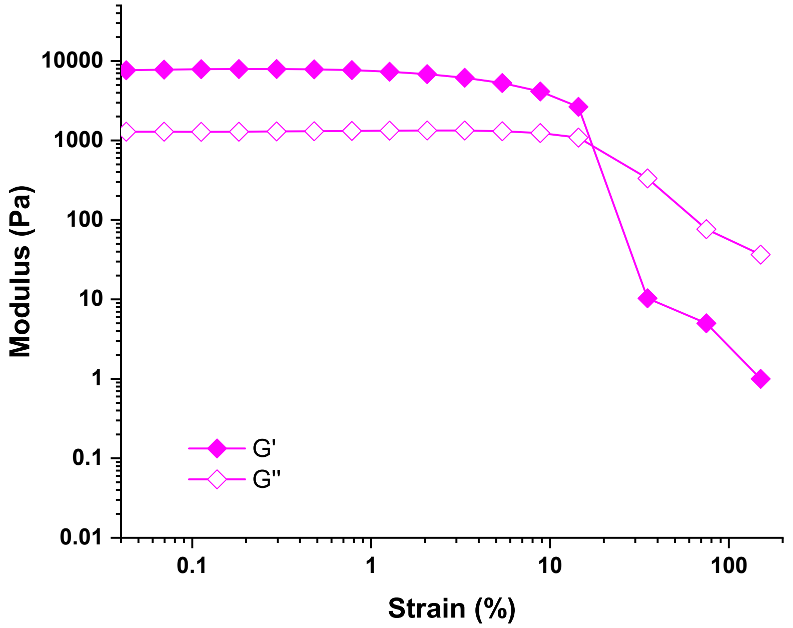

Figure S7. Strain sweep rheological profile of the *in situ* formed Fmoc-(3-Pal)$_2$-NH$_2$ hydrogel in phosphate-buffered saline (pH 7.4).

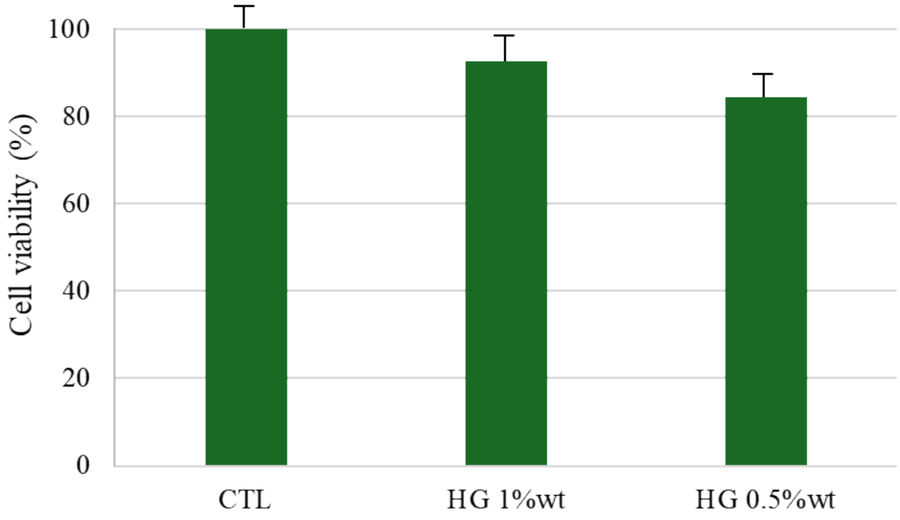

Figure S8: MTT assay was conducted on HEK-293 cells treated for 48 h with hydrogel conditioned media at 1.0 % w/v and at 0.5% w/v. Cell survival was expressed as percentage of viable cells in the presence of conditioned media, compared to control (CTL) cells grown in their absence. Error represents SD of three independent experiments (n.s. = not significant, Mann- Whitney t-test).

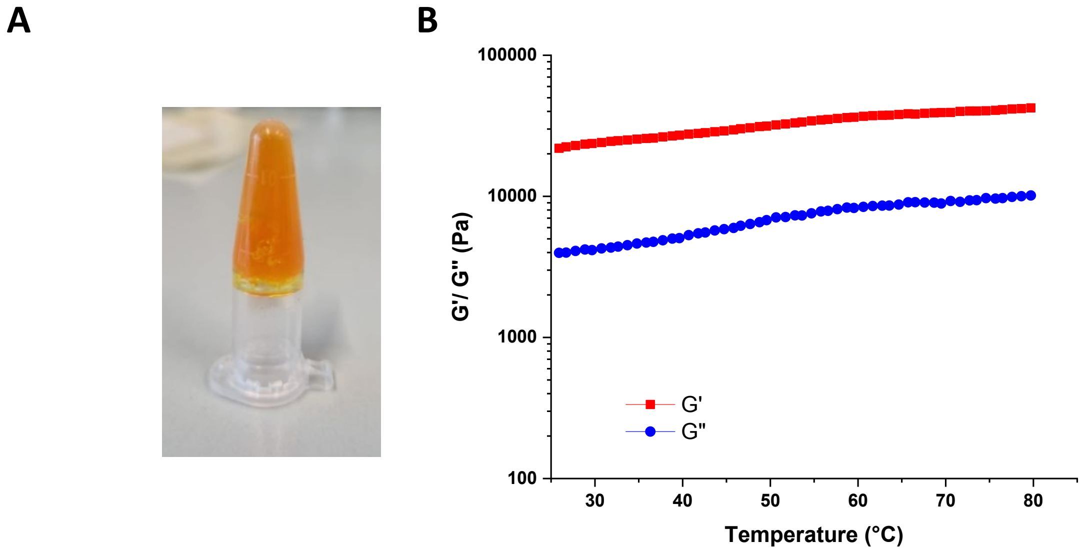

Figure S9. (A) Inverted test tube of the peptide at a concentration 2 % w/v in 0.1 mol L^{-1} PBS at pH 8.0, in presence of 0.2 % w/v of curcumin. **(B)** G' and G'' moduli reported as a function of the temperature increasing from 25°C to 80°C.

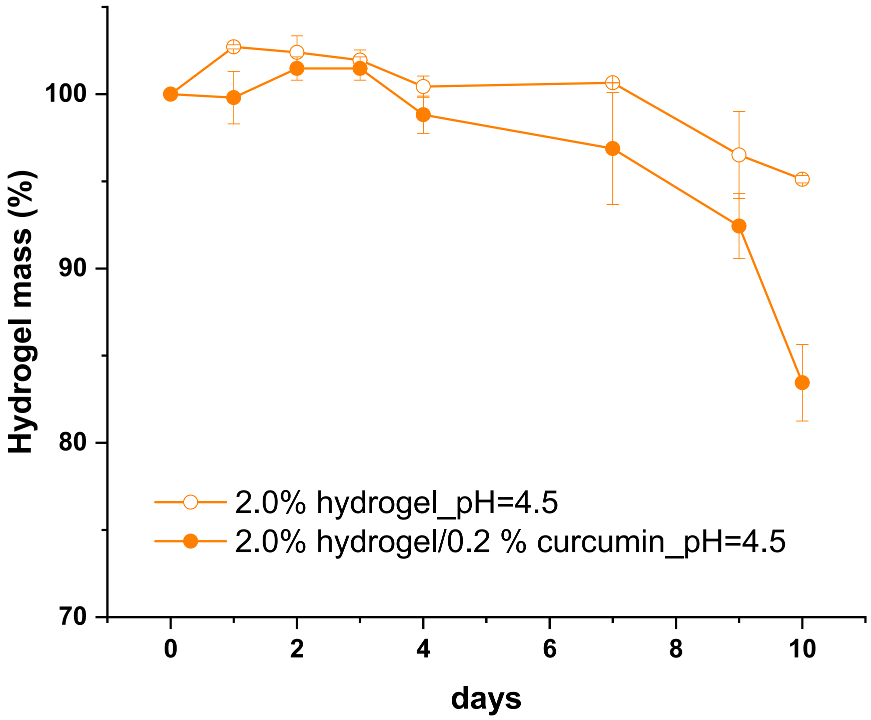

Figure S10. 2.0 % w/v unloaded and curcumin-loaded hydrogel's stability under acidic pH conditions.
